# HBV DNA genome co-transfection procedure for the evaluation of relative fitness

**DOI:** 10.1371/journal.pone.0175543

**Published:** 2017-05-04

**Authors:** Ina Sevic, Maria Mora Gonzalez Lopez Ledesma, Diego Martin Flichman, Rodolfo Hector Campos

**Affiliations:** 1 Universidad de Buenos Aires, Facultad de Farmacia y Bioquímica, Departamento de Microbiología, Inmunología y Biotecnología, Cátedra de Virología, Buenos Aires, Argentina; 2 Consejo Nacional de Investigaciones Científicas y Técnicas (CONICET), Buenos Aires, Argentina; Kliniken der Stadt Köln gGmbH, GERMANY

## Abstract

Hepatitis B virus (HBV) has a high mutation rate and exists as a mixture of genetically different but closely related variants. We present a HBV DNA co-transfection fitness assay and use it to evaluate the relative fitness of different HBV variants in two scenarios: seroconversion process and occupation of an ecological niche. In the seroconversion experiment, subgenotype D1 (sgtD1) deletion (1763–1770) had significantly lower fitness comparing with both sgtD1 wild type and sgtD1mut G1896A, while, in the case of occupation of ecological niche experiment, the results showed the same relative fitness between all of the genotype combinations, except F1b-F4. In this case sgtF1b clearly overgrow sgtF4, which is in accordance with the observation that F1b is the most prevalent in the new infections in Argentina. In summary, we present a method aimed to evaluate HBV viral fitness which improve the analysis of the relative frequency of viral variants during the HBV infection process.

## Introduction

Hepatitis B virus (HBV) is an important global health problem with an estimated 240 million individuals chronically infected worldwide. These patients have a higher probability of developing liver cirrhosis and hepatocellular carcinoma, which causes more than 686 000 deaths annually.[1]

HBV exists in form of quasispecies. Viral quasispecies are the collections of closely related viral genomes subjected to a continuous process of competition among the variants and selection of the fittest variant in a given environment. [2] This process has been difficult to study considering that HBV, in contrast to many other viruses, does not have a cell culture system that allows efficient *in vitro* infection and passaging of virus. The development of an efficient co-transfection method for the evaluation of relative fitness of HBV variants would be a useful tool to improve the knowledge of HBV infection process.

In this work, we develop an efficient HBV DNA genome co-transfection procedure for the evaluation of relative fitness and use it to evaluate the impact of mutations in basic core promoter (BCP) and pre core (pC) regions fixed during the seroconversion process, and the relative fitness of (sub) genotypes (sgt) that are currently prevalent in Argentina.

## Materials and methods

### Serum samples and viral variants

Serum samples were obtained from individuals with well-documented HBV infection. Viral variants that were used in this work were:

Mutated sgtD1 with mutation 1896A (sgtD1mut) and deletion (1763–1770) (sgtD1del).

Wild types (wt): sgtD1, sgtF1b, sgtF4, sgtA2.

All wt and the mutated sgtD1 were naturally occurring variants obtained from the patient’s serum.

Both mutations and all the wild types have been confirmed by direct sequencing of the 3.2-kb fragments that were used for the transfection assays. Full-length genome sequences have been made available in Genbank (KY382412, KY382413, KY382414, KY382415, KY382411, and KY382410).

### DNA extraction and amplification of full length genome

HBV DNA was extracted from 200 μl of serum following phenol-chloroform protocol. Briefly, samples were digested with proteinase K, DNA was extracted with phenol-chloroform and precipitated with isopropanol.

HBV genomes were amplified with the Expand High-Fidelity polymerase (Roche, Germany) using primers with restriction site for the enzyme BspQI (New England Biolabs, USA): HBVs and HBVas. (See Table 1 for PCR cycles and Table 2 for primer details.)

**Table 1:** PCR cycles for full length genome amplification.

	Temperature	Time length	Number of cycles
Initial denaturation	94°C	5 minutes	1
	94°C	40 seconds	
	68°C	3 minutes	
	94°C	40 seconds	10
	59°C	1 minutes	
	68°C	5 minutes	
	59°C	1 minutes	10
	94°C	40 seconds	10
	61°C	1 minutes	
	68°C	7 minutes	
	94°C	40 seconds	10
	61°C	1 minutes	
	68°C	9 minutes	
Final extension	68°C	15 minutes	1

**Table 2:** List of primers used in this study.

Primer	Primer direction	Primer sequence	Position	Restriction enzyme
HBVs	Forward	CCGGAAAGCTTATGCTCTTCTTTTTCACCTAATCATC	1821–1843	BspQI (Underlined Complete restriction site)
HBVas	Reverse	CCGGAGAGCTCATGCTCTTCAAAAAGTTGCATGGTGCTGGTG	1825–1804	BspQI (Underlined Complete restriction site)
HBV1	Forward	GTCAACGACCGACCTTGAGGC	1684–1704	-
HBV2	Reverse	TGAACAGACCAATTTATGCCTACAGCCTCC	1805–1776	-
HBV3	Forward	ATGGAGACCACCGTGAACGC	1608–1627	-
HBV4	Reverse	AATTCTTTATAAGGATCAATGTCCAGGCCC_	1926–1897	NIaIV (Underlined Partial restriction site)
HBV5	Forward	CTGTGCCAAGTGTTTGCTGA	1171–1190	-
HBV6	Forward	CTGCTGGTGGCTCCAGTTC	57–75	-
HBV7	Reverse	AACGCCGCAGACACATCCA	391–373	-
HBV8	Forward	ATTTTACGGGACTCTATTCCTC	2479–2500	-
HBV9	Reverse	GGGACTCAAGATGYTGYACAG	789–769	-

### Cloning and preparation of full length genome for transfection

Following PCR amplification, the 3.2 kb fragments were recovered from the agarose gel and purified using QIAquick Gel Extraction Kit (Qiagen, Germany) and cloned using pGEM-T Easy vector (Invitrogen, USA) according to the manufacturer’s instructions.

For every viral variant 10 to 20 clones (HBV 3.2 kb fragments) were obtained, mixed and grown in order to obtain sufficient quantity for transfection. Vector-HBV DNA was extracted by precipitation with isopropanol. Briefly, for every viral variant a mix of transformed bacteria was grown in liquid medium (LB), bacterial cells were pelleted and pellets were dissolved in a buffer that contained 10 mM EDTA, 50 mM Tris-HCl (pH = 7.5) and 100 μg/ml of RNAse A. Cells were lysed by adding a mix containing 0.2 M NaOH and 1% SDS, and the reaction was neutralized by adding 1.32 M Potassium Acetate (pH = 4.8). Vector-HBV DNA was centrifuged and precipitated with isopropanol.

Full-length linear HBV DNA was released from the plasmid by digestion with BspQI enzyme. The 3.2-kb fragments were gel purified with the QIAquick gel extraction kit and the DNA was then quantified spectrophotometrically.

### Transfection of full length HBV DNA

Huh7 cells were maintained in Dulbecco’s modified Eagle’s medium (DMEM; GIBCO) supplemented with 10% fetal bovine serum and 2.25 g/L NaHCO3. The cells were grown in six-well plates until reaching a density of 70–80% and were transfected with 1μg of full-length HBV DNA mix, composed of 1:1 mass ratio of viral variants that are being confronted. The transfection agent used in these experiments was X-tremeGENE9 (Roche, Germany) according to the manufacturer’s recommendation for 3:1 ratio.

The medium was changed every day, and 96 hours post-transfection the cell culture supernatant was harvested. For every pair of confronted variants two independent experiments were made, and every experiment was performed in triplicate.

### Evaluation of fitness of sgtD1 wt and mutated variants

To evaluate fitness of sgtD1 wt and mutated variants, all combinations were transfected in 1:1 mass ratio. To determine the frequency of each variant in the progeny in all three cases, viral DNA was extracted from the harvested supernatants using commercial nucleic acid extraction kit High Pure Viral Nucleic Acid Extraction kit (Roche, Germany), a PCR was performed and the fragments were cloned as previously described.

For sgtD1 wt vs. sgtD1mut experiment, a 319 bp fragment was amplified using primers HBV3 and HBV4. In order to differentiate these variants, a partial NIaIV restriction site was included with primer HBV4. This primer has only 5 out of 6 nucleotides of NIaIV restriction site. The last nucleotide of the restriction site will be formed after amplification and only if the sequence is wild type. Considering that the wild type in 1896 position has a G, it will make a complete restriction site (G+GGGCC). If the sequence is mutated, the restriction site will not be formed (A+GGGCC). The PCR fragment was digested with NIaIV enzyme (New England Biolabs, USA) and was analyzed on 8% polyacrylamide gel to visualize the 28 nucleotide difference between the digested and non-digested fragment.

In the case of sgtD1del vs. wt and sgtD1del vs. sgtD1mut experiments, the presence of the 8 nucleotide deletion (1763–1770) was determined by a 121bp PCR using primers HBV1 and HBV2, and was analyzed on 15% polyacrylamide gel to visualize the difference between the fragment with (113bp) and without (121bp) deletion.

In all the cases, twenty clones were analyzed for each of the six plates.

(See Table 2 for primer details and Table 3 for PCR cycles, Table 4 for experiment scheme.)

**Table 3:** PCR cycles for subgenomic fragments amplification.

	Temperature	Time length	Number of cycles
Initial denaturation	94°C	5 minutes	1
	94°C	30 seconds	30
	53°C (56°C for HBV6-HBV9; 58°C for HBV1-HBV2)	30 seconds	
	72°C	30 seconds	
Final extension	72°C	5 minutes	1

**Table 4:** sgtD1 wt and mutated variants experiment scheme.

Confronted viral variants	Fragment size	Restriction enzyme	Resulting fragment size
sgtD1 wt + sgtD1mut	320 pb	NIaIV	Wt: 290 pb + 30 pb
			D1mut: 320 pb
sgtD1 wt + sgtD1del	121pb o	-	Wt: 121pb
	113 pb		D1del: 113 pb
sgtD1mut + sgtD1del	121pb o	-	D1mut: 121pb
	113 pb		D1del: 113 pb

### Evaluation of the relative fitness between different genotypes

To evaluate relative fitness between different genotypes, all combinations (F1b vs. F4, F1b vs. A2, F1b vs. D1, F4 vs. A2, F4 vs. D1 and A2 vs. D1) were transfected in 1:1 mass ratio.

To determine the frequency of each genotype in the progeny, in all cases, viral DNA was extracted from all six of the harvested supernatants using commercial nucleic acid extraction kit High Pure Viral Nucleic Acid Extraction kit (Roche, Germany), a PCR was performed and the fragments were cloned as previously described. For each of the six plates, twenty clones were analyzed performing the PCR, digesting the fragment with the corresponding enzyme (see Table 4 for details) and visualizing the difference between the two resulting fragments on 1.5% agarose gel.

To analyze F1b vs. D1 and F4 vs. D1, a PCR fragment of 334bp was digested with Xhol enzyme which has restriction site in sgtD1 but not in F1b or F4. Primers used for this PCR were HBV6 and HBV7.

To analyze F4 vs. A2, a 762bp PCR product was digested with Smal enzyme which has a restriction site in F4 but not in A2 fragment. Primers used for this PCR were HBV6 and HBV9.

To analyze A2 vs. D1, a PCR fragment of 1130bp was digested with EcoRI that has a restriction site in A2 but not in D1 fragment. Primers used to amplify this fragment were HBV7 and HBV8.

In the cases of F1b vs. A2 and F1b vs. F4, a 631bp PCR fragment was digested with HindIII enzyme. HindIII has a restriction site in F1b sequence but not in F4 or A2. Primers used for this reaction were HBV5 and HBV2.

(See Table 2 for primer details and Table 3 for PCR cycles, Table 5 for experiment scheme.)

**Table 5:** Genotype experiment scheme.

Confronted genotypes	Fragment size	Restriction enzyme	Resulting fragment size
D1 + F4	334 pb	Xhol	D1: 264 pb + 70 pb
			F4: 334 pb
D1 + F1b	334 pb	Xhol	D1: 264 pb + 70 pb
			F1b: 334 pb
F1b + F4	631 pb	HindIII	F1b: 463 pb + 168 pb
			F4: 631 pb
A2 + F1b	631 pb	HindIII	F1b: 463 pb + 168 pb
			A2: 631 pb
A2 + F4	762 pb	SmaI	A2: 497 pb + 265 pb
			F4: 762 pb
A2 + D1	1130 pb	EcoRI	A2: 721 pb + 409 pb
			D1: 1130 pb

### Further analysis of the F1b-F4 relationship

Considering that during transfection newly formed viral particles cannot infect other cells, a series of experiments was made in order to evaluate how the dynamic of the progeny change during simulation of second, third and fourth cycle.

After the initial experiment (first cycle) we used the resulting frequencies of F1b and F4 in the progeny as an initial frequency for the next transfection (second cycle), the resulting frequencies of this transfection as an initial frequency for the next one (third cycle), etc.

### Controls

For every experiment, the following parameters were controlled.

One well per experiment was transfected with a mock (HBV not replicative genome, which lacks a part of Core region [2158–2530]) as a control of remaining input.

For every experiment both of the confronted variants have also been transfected separately and analyzed as a control of transfection of that particular DNA mixture. These controls were cloned, multiple clones were selected randomly, amplified and digested in order to verify that every viral variant clones, amplifies and digests as expected.

Also, all the primers used in these experiments were previously evaluated by limit of detection method for the used experimental conditions (number of cycles and temperature of annealing). The results showed that in these conditions the primers amplify confronted variants/subgenotypes with the same efficiency showing the first positive PCR result at the same dilution for both tested variants and with similar PCR product concentration. The amplification was evaluated by agarose gel electrophoresis for both experiments: sgtD1 wt and mutated variants experiment (S1 Fig) and genotype experiment (S2 Fig).

### Statistical analysis

The differences between viral variants were calculated using two sided Fisher’s exact test. The dispersions between repeats were calculated using two sided Chi squared test. A *p*-value of less or equal to 0.05 was considered to indicate statistical significance. All analyses were performed using GraphPad Prism 5.01 software (GraphPad Software, San Diego, CA, USA).

### Ethics Statement

This study was carried out according to the World Medical Association Declaration of Helsinki; it was approved by the Ethics Committee of the School of Pharmacy and Biochemistry, Buenos Aires University (Permit Number: 0069893/2014) and written informed consent statements were signed by all patients.

## Results

### Evaluation of fitness of sgt D1 wt and mutated variants

**Fig 1:**
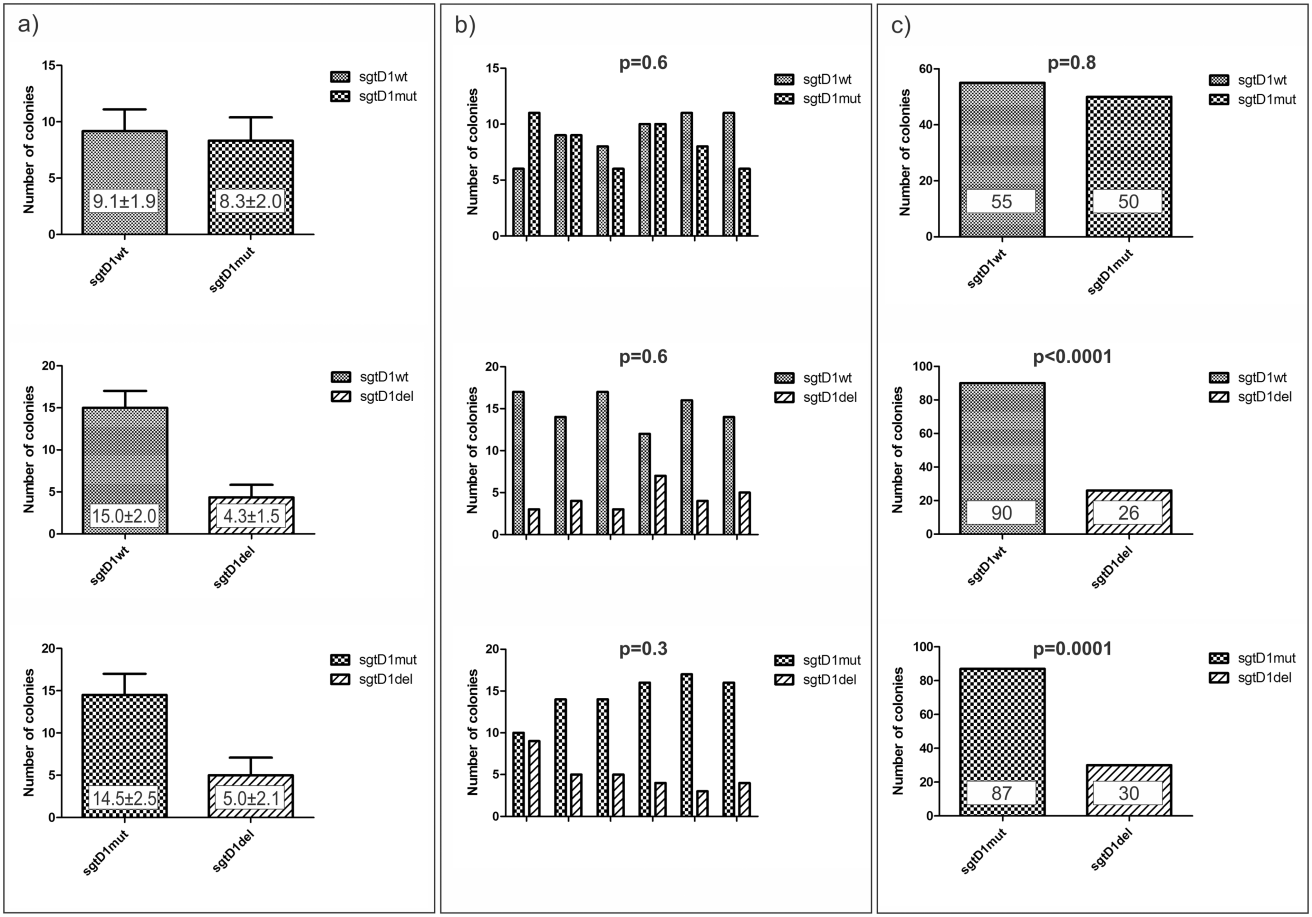
Evaluation of fitness of sgtD1 wt and mutated variants. Huh7 cells were co transfected with two different viral variants. a) Values represent the mean of sextuplicates; error bars represent the calculated standard deviation. b) Bars represent the six repetitions of the experiment; p-value is dispersion between the repetitions calculated using chi-square test. c) The results of the experiments are shown as sums of the repetitions; p-values were calculated using Fisher´s exact test.

The comparative fitness assay among sgtD1, sgtD1del, and sgtD1mut was performed by co-transfection of each pair in 1:1 mass ratio. In the case of the sgtD1 wild type vs. sgtD1mut experiment, the progeny maintained approximately the same ratio as the one used for co-transfection (p = 0.8), while in the cases of sgtD1del vs. sgtD1 wild type or vs. sgtD1mut the resulting progeny was composed mainly of sgtD1 (p<0.0001) or sgtD1mut (p = 0.0001). (Fig 1c)

In all three cases the dispersion between the repetitions gave non-significant p-value. (Fig 1b) The amount of variation is showed with standard deviation. (Fig 1a)

### Evaluation of the relative fitness between different genotypes

Series of experiments were designed to evaluate the potential impact of different fitness values between sub genotypes. To that end, all the possible combinations (F1b vs. F4, F1b vs. A2, F1b vs. D1, F4 vs. A2, F4 vs. D1 and A2 vs. D1) were assayed transfecting the cells in a 1:1 mass ratio.

**Fig 2:**
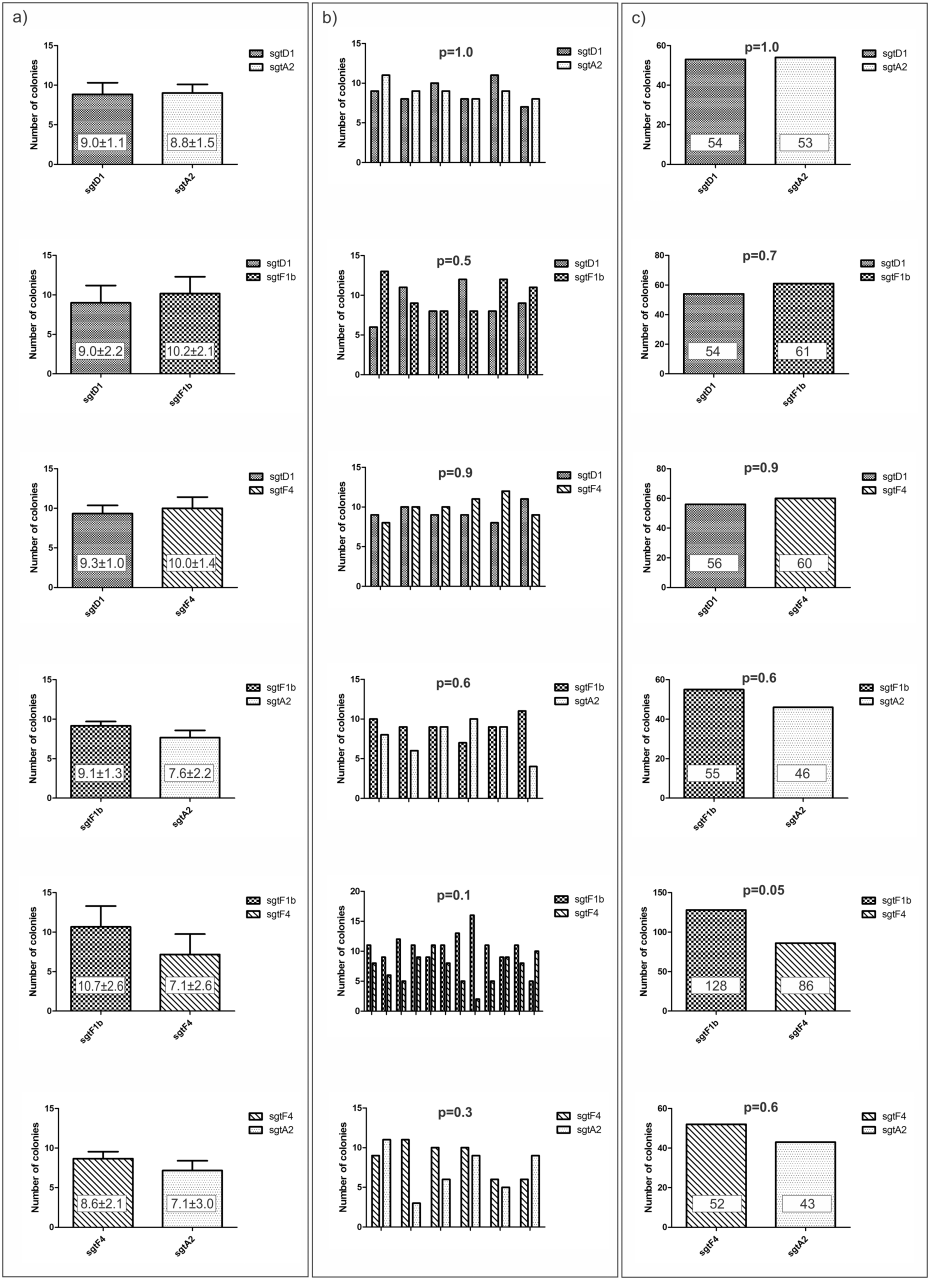
Evaluation of the relative fitness between different genotypes. Huh7 cells were co transfected with two different genotypes. a) Values represent the mean of sextuplicates (12 in the case of F1b-F4 experiment); error bars represent the calculated standard deviation. b) Bars represent the six (12 for F1b-F4) repetitions of the experiment; p-value is a dispersion between the repetitions calculated using chi-squared test. c) The results of the experiments are shown as sums of the repetitions; p-values were calculated using Fisher´s exact test.

In all of the cases, except F1b vs. F4, the progeny maintained approximately the same ratio as the one used for co-transfection, with p-values of 0.7 for sgts F1b vs. D1, 0.9 for sgts F4 vs. D1, 0.6 for sgts F1b vs. A2, 0.6 for sgts F4 vs. A2 and 1.0 for sgts A2 vs. D1. (Fig 2c)

Since the experiment sgtF1b vs. sgtF4 showed a tendency that was near the statistical cut-off, an additional experiment was done in sextuplicate in order to make the statistical analysis more accurate by raising the number of analyzed clones. The resulting progeny presented a higher number of clones of sgtF1b than of sgtF4 with a p-value on the cut-off limit (p = 0.05). (Fig 2c)

In all of the cases the dispersion between the repetitions gave non-significant p-value. (Fig 2b) The amount of variation is showed with standard deviation. (Fig 2a)

### Further analysis of the F1b-F4 relationship

Considering that during transfection newly formed viral particles cannot infect other cells, a series of experiments was made in order to simulate serial reinfections.

After the initial experiment (first cycle) we used the resulting frequencies of sgtF1b and sgtF4 clones of the progeny (60%-40%) as the initial frequency (1.5:1 mass ratio) for the next transfection (second cycle), the resulting frequencies of clones of this transfection (73%-27%) as initial frequency (2.7:1 mass ratio) for the next one (third cycle) and the resulting frequencies (81%-19%) of this transfection as the initial frequency (4.3:1 mass ratio) for the last (fourth) cycle with the resulting frequency 93%-7%. The serial co-transfection experiment showed a clear ability of sgtF1b to overgrow sgtF4 during a competitive viral production assay.

**Fig 3:**
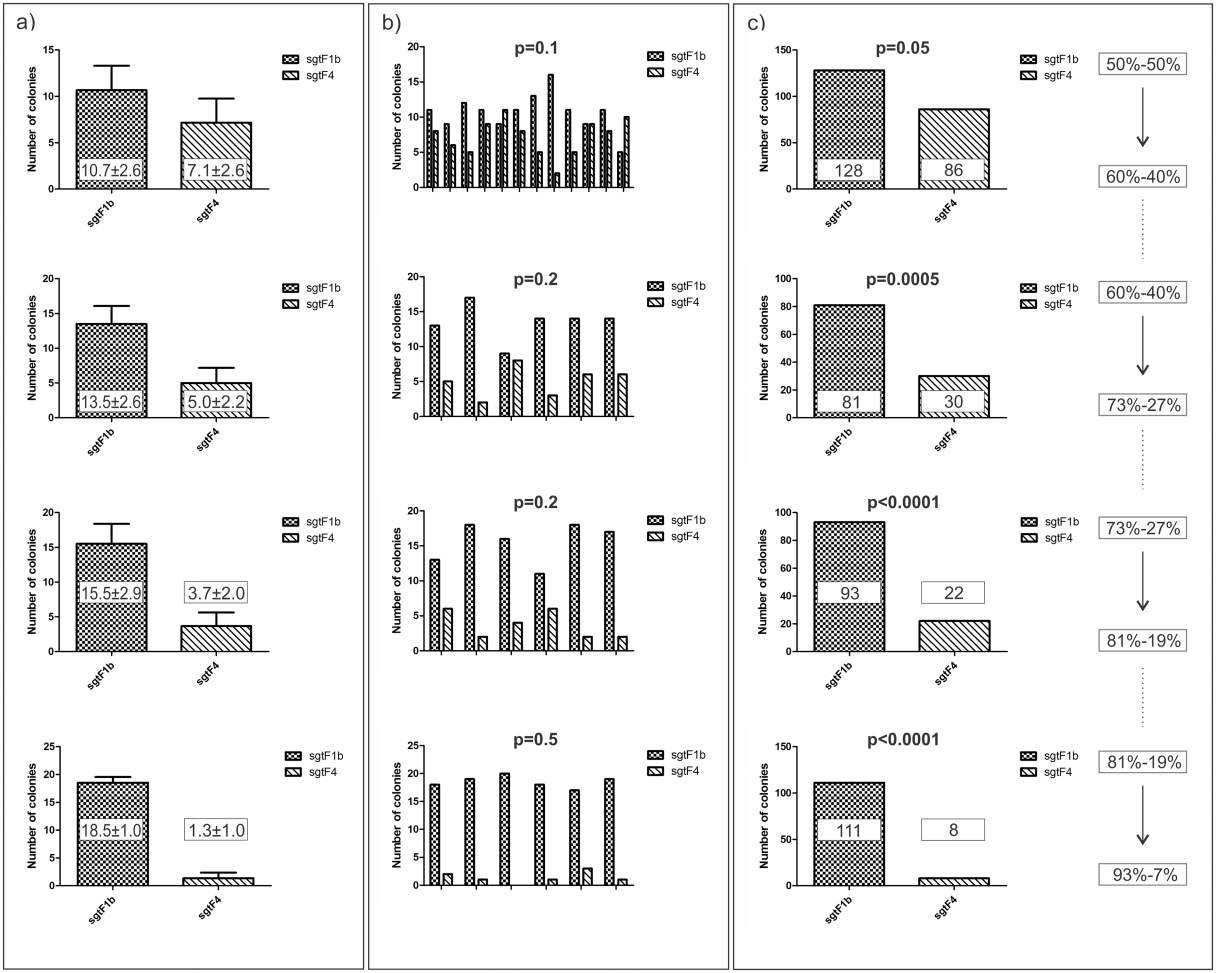
Further analysis of the F1b-F4 relationship. Huh7 cells were co transfected with genotypes F1b and F4. a) Values represent the mean of sextuplicates (12 in the first experiment); error bars represent the calculated standard deviation. b) Bars represent the six repetitions (12 in the first case) of the experiment; p-value is dispersion between the repetitions calculated using chi-square test. c) The results of the experiments are shown as sums of the repetitions; p-values were calculated using Fisher´s exact test.

In all of the cases p-value was significant (Fig 3c), while the dispersion between the repetitions gave non-significant p-values. (Fig 3b) The amount of variation is showed with standard deviation. (Fig 3a)

## Discussion

HBV has a high mutation rate and, as a consequence, the virus exists as a mixture of genetically different but closely related variants whose frequencies depend on the ability of each variant to replicate in a particular environment. If the environment changes, new frequencies of these variants are selected. Occupation of an ecological niche by variants harboring specific mutations or by specific genotypes is a dynamical process that depends on the ability of each viral population to impose over the others in each determined environment.[2,3]

Viral fitness is a relative measure of adaptation of viral variant to one specific environment. The evaluation of the fitness of a given viral variant is of importance for understanding the emergence or the elimination of that variant in a particular microenvironment. [2,4,5]

HBV, in contrast to many other viruses, does not have a cell culture system that allows efficient in vitro infection and passaging of virus, which is a strong constrain for fitness assay. The transfection system is currently widely used for evaluation of different HBV variants. Taking advantage of this system we developed a co-transfection method aiming to simulate a viral fitness assay. After co-transfection with full length genomes of two viral variants we compared the frequencies of these two variants in progeny.

There are many scenarios where the evaluation of fitness would be helpful for understanding the selection process. Here we present a HBV fitness assay and evaluate the relative fitness of different HBV variants in two scenarios: seroconversion process and occupation of an ecological niche.

During seroconversion, mutations in regions of pC and BCP can appear. It has been reported by Sede et al. (2014), after five-year prospective study of a patient, that the three coexisting variants (wild type, sgtD1del and sgtD1mut) maintained similar ratio through seroconversion process, maintaining the deleted viral variant predominant during the entire period.[6] The G1896A mutation in pC region converts tryptophan (TGG) into a translational stop codon (TAG) which stops the production of HBeAg. This mutation occurs most frequently in genotype D and was previously reported to have the same replication level as wild type.[7–9] The 8 nucleotide deletion (1763–1770) can be found in the region of Enhancer II and BCP and is usually associated with low replication and low levels of viral proteins including HBeAg.[10,11] Our results showed that the deleted variant had lower replication levels comparing with both sgtD1 wild type and sgtD1mut, which is in accordance with previous reports [12] but cannot explain by itself the reported behavior in the patient.[6] However, the replication level is not the only factor that determines the selection of one specific viral variant *in vivo*. Other factors, like the selective pressure of the immune system, could explain this observed difference.

HBV presents high heterogeneity and, as a consequence, it has been classified in eight genotypes and multiple subgenotypes. In Argentina, it has been recently reported a high prevalence of sgtF1b in acute and chronic HBeAg positive infections followed by sgtA2, sgtF4 and sgtD.[13–15] Our results showed the same relative fitness between all of the genotype combinations, with the exception of the pair F1b-F4, which showed an increase of sgtF1b in the progeny. Additionally, when serial cycles of infection were simulated, results showed that the ratio between F1b and F4 kept moving in the direction of F1b predominance. This shows a clear ability of sgtF1b to overgrow sgtF4 during the competitive viral production assay. This result is in accordance with the fact that F1b is occupying the ecological niche of new infections in Argentina.[13,16]

There are reports that identify striking differences in replicative capacity and protein expression between different genotypes.[17] Considering that there have been very few studies directly comparing the replication of different HBV genotypes this method could help evaluate some of the observed differences in natural history and disease progression.

In summary, we present a new method aimed to evaluate HBV viral fitness. This method could facilitate the analysis of interactions between different viral variants and could improve the evaluation of comparative fitness *in vitro*, which will in the end contribute to the understanding of the selection mechanisms governing HBV infection.

## Supporting information

S1 Fig Agarose gels showing primer evaluation for the sgtD1 wt and mutated variants experiment. The primers used in these experiments were evaluated by limit of detection method for the used experimental conditions (number of cycles and temperature of annealing). The results showed that in these conditions the primers amplify confronted variants with the same efficiency showing the first positive PCR result at the same dilution for both tested variants and with similar PCR product concentration.
(TIF)

S2 Fig Agarose gels showing primer evaluation for the genotype experiment. The primers used in these experiments were evaluated by limit of detection method for the used experimental conditions (number of cycles and temperature of annealing). The results showed that in these conditions the primers amplify confronted subgenotypes with the same efficiency showing the first positive PCR result at the same dilution for both tested variants and with similar PCR product concentration.
(TIF)
