# Supplementary figures and images for: HBV DNA genome co-transfection procedure for the evaluation of relative fitness
Source: PLoS One. 2017 May 4;12(5):e0175543. doi: 10.1371/journal.pone.0175543 (PMC5417490; doi:10.1371/journal.pone.0175543)

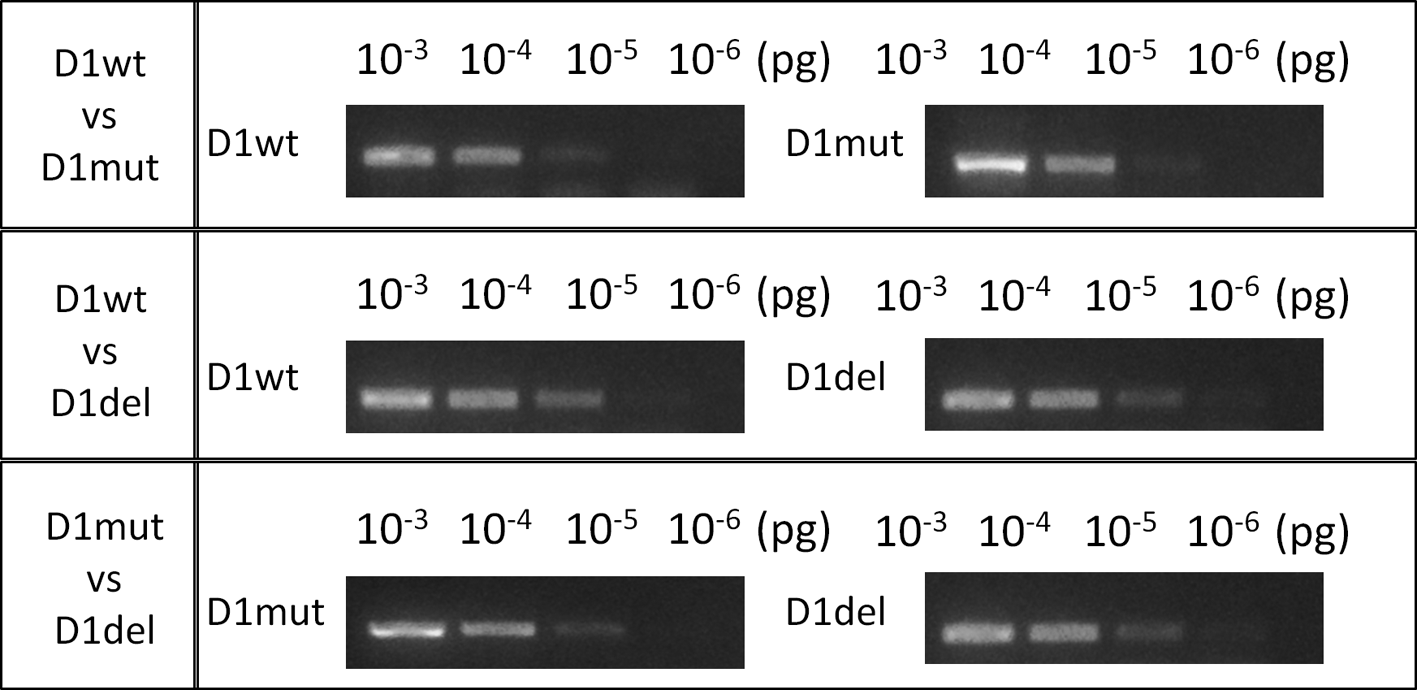

Supplement: S1 Fig — The primers used in these experiments were evaluated by limit of detection method for the used experimental conditions (number of cycles and temperature of annealing). The results showed that in these conditions the primers amplify confronted variants with the same efficiency showing the first positive PCR result at the same dilution for both tested variants and with similar PCR product concentration. (TIF) [file pone.0175543.s001.tif]

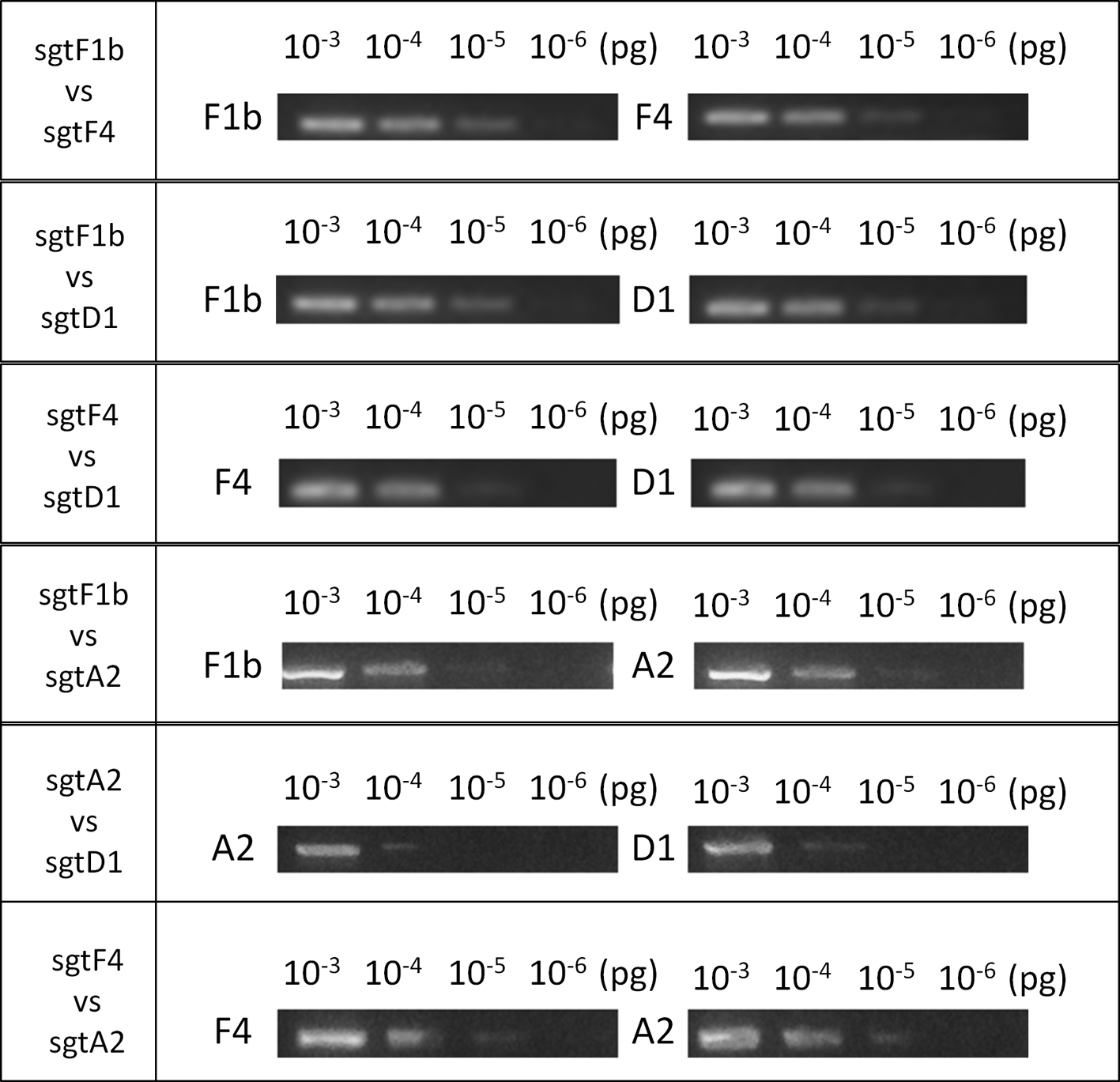

Supplement: S2 Fig — The primers used in these experiments were evaluated by limit of detection method for the used experimental conditions (number of cycles and temperature of annealing). The results showed that in these conditions the primers amplify confronted subgenotypes with the same efficiency showing the first positive PCR result at the same dilution for both tested variants and with similar PCR product concentration. (TIF) [file pone.0175543.s002.tif]
